# Antioxidant Potential and Volatile Aroma Profiling of Red Wines from the Tarnave Vineyard

**DOI:** 10.3390/molecules30193853

**Published:** 2025-09-23

**Authors:** Diana Ionela Popescu (Stegarus), Wilhemine Claudia Nicoleta Sas, Ovidiu Tița, Violeta-Carolina Niculescu, Nicoleta Anca Ionescu (Șuțan)

**Affiliations:** 1 National Research and Development Institute for Cryogenic and Isotopic Technologies—ICSI Ramnicu Valcea, 4th Uzinei Street, 240050 Ramnicu Valcea, Romania; diana.stegarus@icsi.ro; 2 Doctoral School, Lucian Blaga University of Sibiu, Victoriei Street No. 10, 550024 Sibiu, Romania; 3 Faculty of Agricultural Sciences, Food Industry and Environmental Protection, Lucian Blaga University of Sibiu, Dr. Ion Rațiu Street, No. 7–9, 550012 Sibiu, Romania; ovidiu.tita@ulbsibiu.ro; 4 Department of Natural Sciences, National University of Science and Technology POLITEHNICA Bucharest, Pitesti University Centre, 1st Targu din Vale Street, 110040 Pitesti, Romania; nicoleta_anca.sutan@upb.ro

**Keywords:** antioxidant activity, GS-MS, polyphenols, red wines, volatile compounds

## Abstract

The increasing demand for red wines, supported by their complex sensory features and rich biochemical composition, has encouraged cultivation in non-traditional viticultural regions. This study investigates the antioxidant potential and volatile composition of three red grape cultivars (Feteasca neagra, Merlot, and Pinot noir) cultivated in the Tarnave Vineyard, Romania, a region historically dedicated to white wines but now increasingly favorable to red varieties due to climate change. Antioxidant capacity, assessed via DPPH, Trolox equivalent antioxidant capacity (TEAC), and Ferric reducing antioxidant power (FRAP) assays, identified Feteasca neagra as the most potent (IC_50_: 115.32 µg/mL; FRAP: 13.45 mmol TE/L). Gas chromatography–mass spectrometry (GC–MS) profiling identified 61 volatile compounds, with Pinot noir showing the highest concentration (99,018.57 µg/L). Multivariate analysis (ANOVA, PCA) confirmed significant varietal differences and terroir-specific influences on wine composition. Pinot noir was characterized by high levels of higher alcohols, esters, and lactones, yielding a floral and fruity aroma, while Feteasca neagra exhibited intense color, high flavonoid content (notably malvidin-3-glucoside), and vanilla–herbal notes. Merlot presented a balanced sensory profile with significant phenolic acid content. These findings highlight the chemical and sensory potential of the Tarnave Vineyard for premium red wine production.

## 1. Introduction

Climate change significantly affects the quality and composition of red grapes by altering the biosynthesis of sugars, phenolic compounds (including polyphenols and anthocyanins), organic acids, and volatile aroma constituents [1]. Recent global and European climatic shifts have facilitated the acclimatization of grape varieties previously unsuited to certain regions. Red grape cultivars, in particular, require specific pedo-climatic conditions to attain optimal viticultural value, with soil and climate being decisive factors [2,3,4].

Moderate temperature strengthens photosynthesis and accelerates ripening, leading to elevated glucose and fructose concentrations and, consequently, to higher potential alcohol levels in the must. On the contrary, extreme heat reduces tartaric and malic acid levels, affecting sensory balance, accelerating anthocyanin degradation, and diminishing the synthesis of key aroma compounds, especially terpenes and norisoprenoids. Although increased solar radiation may stimulate the production of polyphenols and tannins, excessive exposure can disrupt phenolic balance and result in aroma compound losses [5,6,7]. Water availability is equally critical. Moderate water deficits and drought conditions can concentrate bioactive compounds, increasing sugar and polyphenol levels. However, severe water stress impairs photosynthesis and volatile compound synthesis, ultimately degrading grape and wine quality. Excessive rainfall, particularly near harvest, may dilute sugars, anthocyanins, and tannins, while also elevating the risk of fungal infections [8,9].

The aromatic potential of red grape varieties can yield wines of exceptional quality, characterized by regional typicity and varietal distinctiveness, with rich bioactive compounds, which contribute to complex analytical profiles and are associated with potential health benefits [10,11,12,13,14].

Extensive research highlights the influence of terroir, with each region imparting distinct characteristics to grapevines and wines. Temperate climates support high-quality viticulture, while additional factors *topography, hydrology, solar exposure, slope gradient, geological substrate, and airflow) shape the chemical profile of grapes. The accumulation of key compounds, including phenolics, volatile organic compounds (VOCs), and organic acids, quantitatively reflects these site-specific environmental influences [15,16,17].

Volatile profiles are also influenced by raw materials and winemaking [18,19], some volatile compounds serving as authenticity markers by reflecting natural origin of ingredients and unique production methods [20]. Furthermore, geographical origin can be ascertained through the volatile profile, serving as a biochemical fingerprint by capturing terroir-driven variations in soil composition, climate, and agronomic practices that influence volatile compound synthesis [21,22,23,24,25,26,27,28,29,30,31,32].

According to the most recent global climate assessments, 2023 was the warmest year on record, with global average surface air temperatures reaching approximately 1.45 ± 0.12 °C above the pre-industrial baseline (1850–1900), underscoring the accelerating pace of climate change [33]. These increases are consistent with broader trends of rising growing season temperatures, altered precipitation regimes, and increased frequency of extreme climatic events reported across Europe. Such changes have direct implications for viticulture, influencing phenological development, grape ripening dynamics, and aroma compound biosynthesis. Within this global framework, the climatic evolution in 2023 in the region of Tarnave Vineyard can be contextualized as part of a wider pattern in which regions traditionally considered marginal for red grape varieties are becoming increasingly suitable for their cultivation.

Red wines contain significant concentrations of polyphenols, which play an essential role in defining their taste, color, and health-promoting properties, aligning with the volatile compounds present in these products [34,35,36,37,38]. Polyphenols concentrations and their antioxidant potential are critically influenced by grape variety, winemaking techniques, and aging duration [39,40]. These compounds are predominantly derived from grape skins, seeds, and stems and are critical for sensory complexity and oxidative stability. The most prevalent polyphenols belong to the flavonoid and non-flavonoid classes, both of which significantly influence wine quality [41]. Flavonoids include anthocyanins (pigments responsible for the characteristic red–violet to red–brown hues in red wines, influenced by grape maturity), flavonols (which modulate yellow and golden nuances in white wines), and tannins (imparting astringency and structural complexity). These compounds are predominantly localized in grape skins, seeds, and oak barrels used during wine aging. Anthocyanins are water-soluble pigments responsible for the red, violet, and blue coloration of fruits and flowers. Common anthocyanins include cyanidin, delphinidin, and malvidin [42]. These compounds demonstrate sensitivity to light, pH, and temperature, factors that may accelerate their degradation during storage or processing [43]. Their concentration is influenced by grape cultivar and oenological practices [44,45]. During wine aging, anthocyanins may undergo polymerization with tannins, forming stable pigmented polymers that enhance chromatic stability [46]. Non-flavonoids comprise phenolic acids (e.g., gallic, caffeic, and coumaric acids) that enhance oxidative stability and flavor profiles, as well as stilbenes, most notably resveratrol, recognized for its antioxidant properties and cardioprotective benefits [47,48].

Red wines exhibit robust antioxidant activity due to their high concentrations of polyphenols and anthocyanins. Recent research has further clarified their health-promoting potential. For example, a comprehensive review found that grape polyphenols exhibit strong antioxidant and anti-inflammatory effects, modulate gut microbiota, improve insulin sensitivity, and offer cardioprotective benefits by reducing LDL oxidation and improving endothelial function [49,50]. Another study from Romania (2023) showed that phytochemical content and flavonoid and phenolic fraction in red grape varieties correlate strongly with antioxidant activity, suggesting their utility as functional food components [51]. Moreover, a recent animal model experiment demonstrated that consumption of red grapes influenced adipose tissue metabolic markers: grapes rich in anthocyanins increased energy expenditure and upregulated browning markers in white adipose tissue, effects which are considered beneficial in obesity prevention and metabolic health [52]. These findings underscore the relevance of examining bioactive compounds not only for wine quality and sensory properties, but also for their potential human health implications.

Several red grape cultivars have been successfully acclimatized in regions historically deemed unsuitable for their cultivation and ripening. Under current climate warming trends, select cultivars have adapted, yielding wines with distinctive aromatic profiles and enhanced gustatory complexity [53,54,55,56].

This study aims to establish reference benchmarks for phenolic content, anthocyanin levels, and antioxidant capacity in three red wine varietals (Feteasca neagra, Merlot, and Pinot noir) cultivated from grapes acclimatized to the Tarnave Vineyard, a region traditionally known for white wines but currently undergoing significant climatic shifts. By combining spectrophotometric, chromatographic, and advanced statistical methods, the research evaluates the oenological viability of these red wines under changing climate conditions. A novel aspect of this work is the detailed characterization of volatile compound profiles, which enables an empirical connection between biochemical composition and sensory attributes. This approach offers a robust framework for authenticating regional wine typicity and assessing market potential in an evolving viticultural landscape.

This research represents the first comprehensive phenolic and aromatic profiling of red wines from the Tarnave Vineyard, providing critical insights into successful red grape cultivation in a temperate zone previously considered unsuitable. These findings bridge agronomic data with practical strategies for vineyard management and market positioning, thus advancing climate-resilient viticulture and positioning these wines competitively alongside established wine regions.

## 2. Results and Discussion

### 2.1. Climatic Parameter Characterization for the Mica Vineyard Region

To assess the viability of cultivating red varietals in the Tarnave Vineyard, a comprehensive analysis was conducted on the temporal trends of key bioclimatic indicators. These indicators, which delineate the agroclimatic suitability for viticulture, were evaluated over the period 1950–2023. Table 1 synthesizes their mean values, offering a systematic representation of climatic evolution and its implications for viticultural adaptation in the region.

The analysis of the mean values of climatic indicators across five distinct temporal intervals (1950–1963, 1964–1978, 1979–1993, 1994–2008, 2009–2023) reveals a statistically significant upward trend in global solar radiation (MJ m^−2^ day^−1^), increasing from 20.56 MJ m^2^/day^1^ during the 1950–1963 to 22.24 MJ m^2^/day^1^ in 2009–2023.

The effective temperature sum exhibited moderate inter-decadal fluctuations but demonstrated a cumulative increase from 1533.32 °C (1950–1963) to 1612.47 °C (2009–2023), along with the RHI increase from 1.79 to 2.01 and the I_bcv_ significant increase from 7.42 to 9.12.

The HTC displayed temporal variability, reaching maximum values at 1.78 (1964–1978) and 1.77 (1979–1993), followed by a decrease to 1.32 (2009–2023). On the contrary, the DMI increased from 17.43 to 19.96 and simultaneously, the IAOe rose from 29.22 to 32.67.

Because of climate change, mainly rising average annual temperatures and increased summer daily temperatures, efforts have been made to acclimate red grape varieties that previously held limited agronomic interest in the region.

Climatic indicators revealed a 7.9% rise in global solar radiation (1950–2023), enhancing photosynthetic activity and biomass accumulation [54,57]. The effective temperature sum positively correlated with grape berry maturation, driven by elevated enzymatic activity for sugar synthesis and phenolic development [58]. However, such warming trends may concurrently elevate edaphic evapotranspiration rates, potentially altering soil moisture regimes and necessitating adaptive irrigation strategies in viticultural systems [59,60].

The increasing RHI reflected a statistically significant enhancement in thermic resource availability for grapevine cultivation. Its upward trajectory revealed improved agroclimatic suitability, as the RHI quantifies the synergistic balance between cumulative thermal energy and solar radiation during critical phenological stages. A value exceeding 2.0 indicated conditions for late-ripening varieties, underscoring a regional shift toward warmer bioclimatic regimes that may optimize photosynthetic efficiency and metabolic processes in viticultural systems. The trend aligned with improved thermal and radiative conditions, favoring the cultivation of late-ripening red grape varieties, which requires extended growing seasons and elevated heat accumulation [61,62,63].

Climatic indicators suggested a progressive aridification, probably driven by increased temperatures and altered precipitation regimes, which may exacerbate soil moisture deficits and necessitate adaptive water management strategies. The regional shift toward drier conditions was further corroborated by the increase in DMI. While this index typically quantifies aridity via precipitation-to-temperature ratios, the observed increase paradoxically aligns with localized aridification patterns, potentially reflecting increased climatic variability or methodological nuances in regional parameterization. Stomatal closure intensified with soil drying, revealing cultivar-specific sensitivities. Grapevines mitigated water loss by activating adaptive physiological responses, including abscisic acid-induced stomatal closure, accumulation of osmolytes to preserve cellular hydration, drought priming (enhanced stress memory), and potential epigenetic transformations. Together, these mechanisms optimized water-use and limited transpiration under prolonged drought conditions [64,65]. Therefore, climate change-induced water deficits in vineyards may require understanding physiological mechanisms across *V. vinifera* cultivars to optimize water management and cultivar selection.

Improved climatic conditions for producing premium-quality wines, including those derived from red grape varietals was highlighted by the elevated IAOe values correlated with optimized phenological synchronization, enhancing sugar-to-acid balance and phenolic maturity in fruits [66,67].

The red wines produced within this delineated terroir demonstrated distinctive typicity and geographical specificity, resulting from the synergistic interaction of pedoclimatic conditions (mesoclimatic variability, soil geochemical properties, and site-specific ecological dynamics), that significantly differentiate them from wines produced in neighboring viticultural areas. The quantified parameters (Table 1) generate forensic datasets essential for geographical authentication, allowing the validation of wine origin through chemometric profiling (trace element ratios and stable isotope δ^13^C/δ^18^O signatures) and facilitating fraud detection by identifying deviations from established typicity benchmarks, including unauthorized blending or non-compliant viticultural routines.

### 2.2. Polyphenolic Composition and Antioxidant Implications

Total polyphenol concentrations (Table 2) exhibited a descending hierarchy: Feteasca neagra (2814.22 mg GAE/L) > Pinot noir (2539.19 mg GAE/L) > Merlot (2328.34 mg GAE/L).

Data were expressed as the mean of triplicate measurements ± standard deviation; mg GAE/L = milligrams of gallic acid equivalent per liter; mg M3GE/L = milligrams of malvidin-3-glucoside equivalent per liter; DPPH = 1,1′-Diphenyl-2-picrylhydrazyl radical; IC_50_ (µg/mL) = antioxidant efficiency expressed as the half-maximal inhibitory concentration (µg/mL); mmol TE/L = millimoles of Trolox equivalent per liter; TEAC (mmol TE/L) = Trolox equivalent antioxidant capacity, expressed as millimoles of Trolox equivalent per liter (mmol TE/L); FRAP (mmol TE/L) = Ferric reducing antioxidant power, expressed as millimoles of Trolox equivalent per liter (mmol TE/L).

The DPPH (2,2-diphenyl-1-picrylhydrazyl) and ABTS (2,2-azino-bis-3-ethylbenzothiazoline-6-sulphonic acid) assays are widely employed to quantify antioxidant capacity, with grape varietal selection, fermentation conditions, and maturation protocols significantly modulating this property [68,69,70].

The DPPH radical scavenging capacity was lowest in Merlot wine (68%), corresponding to an IC_50_ value of 228.12 µg/mL. In contrast, Feteasca neagra exhibited a significantly lower IC_50_ (115.32 µg/mL).

TEAC and FRAP assays corroborated the trends observed in DPPH and IC_50_ metrics. Both methods validated the superior antioxidant capacity of Feteasca neagra, with TEAC maximum value of 13.62 mmol TE/L, followed by Pinot noir (12.02 mmol TE/L) and Merlot (approximately 3% lower than Pinot noir). Feteasca neagra dominated across all metrics, with polyphenols of 2814.22 mg GAE/L; anthocyanins—526.33 mg M3GE/L; TEAC—13.62 mmol TE/L.

Polyphenols enhance antioxidant properties, chromatic stability, and organoleptic complexity in wines [40]. Feteasca neagra exhibits superior total polyphenol concentrations (~500 mg GAE/L vs. Merlot), correlating with enhanced oxidative aging potential and nutritional merit [71,72]. Pinot noir displays intermediate values, balancing bioactive richness and gustatory subtlety, likely due to varietal-specific phenolic metabolism.

Feteasca neagra’s significantly lower IC_50_ (115.32 µg/mL vs. Merlot: 228.12 µg/mL) and superior DPPH inhibition confirm its antioxidant efficiency, attributed to flavonoid derivatives (e.g., quercetin, catechin oligomers) that synergistically enhance redox activity [73]. FRAP results confirmed the hierarchy observed with DPPH and TEAC assays, underlining the contribution of phenolic acids and anthocyanins to antioxidant capacity. A statistically significant positive correlation (*p* < 0.05) was observed between total polyphenol content and antioxidant capacity, as measured by TEAC, FRAP, and DPPH assays. The inverse proportionality of IC_50_ values underscores the efficiency of polyphenol-rich matrices in oxidative stress mitigation, aligning with mechanisms where anthocyanins stabilize ROS via electron donation, while tannins prolong antioxidant effects and hinder color degradation [74,75].

Current research provides critical insights into the variability of polyphenol-antioxidant relationships across red wine cultivars, with distinct quantitative profiles observed for each varietal. For instance, Rebenciuc et al. [37] identified total polyphenol concentrations in Romanian Feteasca neagra wines ranging from 967 mg GAE/L to 1004 mg GAE/L; Büyüktuncel et al. [76] quantified total polyphenols in eight Turkish red wine varieties between 2599.90 mg GAE/L and 4846.57 mg GAE/L, with antioxidant activity, evaluated via DPPH, ABTS, FRAP, and CUPRAC assays, spanning 7.49–15.93 mmol/L, 12.02–24.73 mmol/L, 12.65–27.68 mmol/L, and 13.19–31.07 mmol/L, respectively; Romano et al. [77] investigated yeast strain impacts on polyphenol accumulation and antioxidant activity in red wines, reporting total polyphenols between 600 mg GAE/L and 790 mg GAE/L and TEAC values of 63–65 nmol TE/mL; Radeka et al. [78] analyzed four Croatian red wines, documenting total polyphenols from 1500 mg GAE/L to 3800 mg GAE/L, FRAP activity between 18 mM Fe^2+^ and 39 mM Fe^2+^, and ORAC values of 22–50 mM Trolox. Onache et al. [79] observed total polyphenol concentrations of 2192–2937 mg GAE/L in Romanian red wines, with antioxidant activity ranging from 14.29 mmol TE/L to 15.62 mmol TE/L.

Analysis of the three red wines revealed significant differences in their anthocyanin content, with Feteasca neagra demonstrating the highest concentrations, which correlated with its superior antioxidant activity. While this study quantifies the total anthocyanin content, future research could delve into a more detailed structural analysis to elucidate the relationship between specific anthocyanin profiles and the long-term color and functional stability of the wines. Total anthocyanin concentrations followed a varietal gradient like polyphenols: Feteasca neagra (526.33 mg M3GE/L) > Pinot noir (477.77 mg M3GE/L) > Merlot (423.32 mg M3GE/L). The anthocyanin-to-polyphenol ratio in Feteasca neagra (0.187) and Pinot noir (0.188) indicated a higher proportional contribution of anthocyanins to total phenolics compared to Merlot (0.181), amplifying their role in both aesthetic appeal and free radical scavenging efficacy.

### 2.3. Volatile Compounds Quantification

The identified compounds align with findings in specialized literature, with comparable values reported in various studies [15,80,81]. For instance, Niculescu et al. [2] documented concentrations of 29,756.97 µg/L volatile compound for Feteasca neagra and 29,545.98 µg/L for Pinot noir, while Manolache et al. [82] reported total volatile compound levels of 58,446.22 µg/L for Merlot, 48,331.23 µg/L for Pinot noir, and 45,655.60 µg/L for Feteasca neagra, depending on the number of identified compounds. These parallels underscore the robustness of the observed trends, though variations in absolute values likely reflect methodological or regional differences. The mean differences in key compounds (e.g., 2-phenylethanol, ethyl acetate) highlight their role as biomarkers for varietal distinction, with their sensory impacts corroborating established biochemical pathways.

A total of 61 volatile compounds were identified and quantified, significantly contributing to the sensory and olfactory profiles of Merlot, Pinot noir, and Feteasca neagra from Tarnave Vineyard.

**Figure 1:**
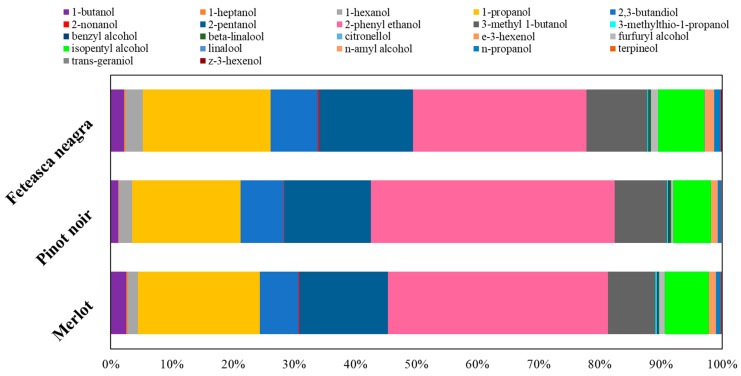
Alcohols’ compositional profiles (%).

The alcohols accumulated at maximum levels in Pinot noir wine (49,275.68 µg/L), followed by Merlot (39,149.59 µg/L) and Feteasca neagra (38,785.77 µg/L) (Figure 1).

Higher alcohols generally result from the catabolism of amino acids during fermentation and may significantly contribute to the sensory profile of wine, mainly to its fusel, alcoholic, and fruity notes [83,84,85,86,87,88,89,90,91,92,93]. Among them, 1-propanol was the most abundant compound across all samples, ranging from 7789.92 ± 41.95 µg/L in Merlot to 8753.67 ± 43.23 µg/L in Pinot noir. This may indicate a strong contribution to the alcoholic aroma, particularly in Pinot noir. 3-methyl-1-butanol (isoamyl alcohol), known for its banana and solvent-like odor, exhibited the highest concentration in Pinot noir (4211.55 ± 33.66 µg/L), followed by Feteasca neagra and Merlot, enhancing the fermented and fruity perception of this variety.

Isopentyl alcohol, n-amyl alcohol, and 1-butanol also had significant levels, with a maximum value in Merlot (987.21 ± 20.11 µg/L), potentially contributing to a more pronounced fusel feature [89]. 1-hexanol, a compound associated with green and herbaceous aromas [83], was notably higher in Feteasca neagra (1111.04 ± 25.02 µg/L), suggesting a more vegetal bouquet.

2-Phenylethanol, a compound with a rose-like, floral aroma [84], was the most abundant aromatic alcohol identified, with the highest concentration found in Pinot noir (19,672.21 ± 71.59 µg/L), followed by Merlot and Feteasca neagra. This compound is typically associated with desirable floral notes and strengthens the elegance and complexity of wine aroma. The elevated level in Pinot noir indicated a strong floral component in its aromatic profile.

Benzyl alcohol, providing mild candy or fruity aroma [85], showed comparable concentrations across the varieties, with a slight predominance in Pinot noir (108.78 ± 6.65 µg/L). 3-methylthio-1-propanol, a sulfur-containing alcohol associated with meaty or onion-like aromas [82], was highest in Merlot (89.63 ± 9.43 µg/L), adding a spicy or umami fragrance to its profile.

2,3-Butanediol, a fermentation-derived diol with limited aroma but notable influence on wine viscosity and mouthfeel [86], varied from 2475.82 ± 31.21 µg/L in Merlot to 3471.14 ± 28.57 µg/L in Pinot noir. 2-pentanol followed a similar pattern, with the highest level again in Pinot noir (7001.55 ± 40.98 µg/L), indicating a fermentation profile that could contribute to smoother mouthfeel and subtle fruity notes. 2-nonanol, an alcohol with a waxy or green-fruity aroma [83], had the highest concentration in Feteasca neagra (78.47 ± 0.48 µg/L), enriching the body and aromatic roundness of the wine.

Terpenic alcohols are generally vital to the floral and citrus components of wine aroma [85,87,91]. Among them, linalool, beta-linalool, citronellol, terpineol, and trans-geraniol were identified. Feteasca neagra exhibited elevated levels of linalool (15.01 ± 1.52 µg/L), beta-linalool (105.34 ± 6.88 µg/L), and trans-geraniol (17.11 ± 0.01 µg/L), suggesting a more complex and floral-citrus aroma compared to the other varieties. Citronellol was undetectable in Pinot noir but was present in Merlot (12.33 ± 0.03 µg/L) and Feteasca neagra (4.24 ± 0.06 µg/L), potentially impacting the perception of freshness and floral fragrance.

Furfuryl alcohol, a compound with roasted and caramel-like notes typically formed during fermentation or aging [94], was most abundant in Feteasca neagra (441.01 ± 11.15 µg/L), reflecting a stronger toasted or oxidative character.

Green leaf volatiles such as z-3-hexenol and e-3-hexenol, contributing to herbaceous and fruity notes [95], had maximum levels in Feteasca neagra (93.09 ± 16.84 µg/L and 5.88 ± 0.39 µg/L, respectively), enhancing its vegetal and fresh character.

**Figure 2:**
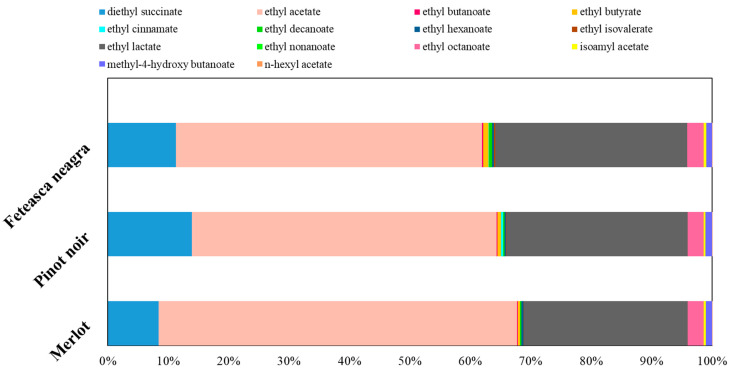
Esters’ compositional profiles (%).

Esters are considered among the most important volatile compounds in wines due to their low odor thresholds and strong effects on fruity and floral aromas [95,96,97,98]. They mainly result during alcoholic fermentation through enzymatic reactions between alcohols and organic acids. Pinot noir wine exhibited significantly higher ester accumulation (26,289.79 µg/L), followed by Merlot (24,435.97 µg/L) and Feteasca neagra (21,562.37 µg/L) (Figure 2).

Ethyl acetate was the most abundant ester from all samples, varying from 15,254.14 ± 96.76 µg/L in Feteasca neagra to 19,234.01 ± 95.01 µg/L in Merlot. High concentrations of ethyl acetate can lead to solvent-like or vinegary off-notes; however, moderate levels are usually associated with pleasant fruity aromas. The relatively increased concentration in Merlot may enhance its more fruity and volatile bouquet [83], though sensory thresholds should be evaluated together with the potential sensory impact.

Ethyl lactate, another abundant ester, was most concentrated in Pinot noir (10,833.21 ± 80.69 µg/L), followed by Feteasca neagra and Merlot. This compound is generated through malolactic fermentation and contributes to a buttery or subtle fruity aroma, indicating a softer and more rounded mouthfeel in Pinot noir [93].

Esters such as ethyl hexanoate, ethyl octanoate, and ethyl decanoate are correlated with green apple, pineapple, banana, and fatty–fruity aromas [83,87]. Among them, ethyl octanoate had the highest concentration in Pinot noir (944.77 ± 44.47 µg/L), followed closely by Merlot and Feteasca neagra. Ethyl decanoate was abundant in Feteasca neagra (138.39 ± 17.41 µg/L), indicating a slightly richer and waxier fruit fragrance. Ethyl hexanoate, linked with apple and green fruit aromas [83], had the highest level in Merlot (112.54 ± 7.13 µg/L). Feteasca neagra also showed increased levels (102.18 ± 8.64 µg/L), contributing to a bright fruity perception.

Isoamyl acetate, imprinting banana, and pear-like notes [93,97], was highest in Feteasca neagra (133.47 ± 9.99 µg/L), compared to Pinot noir and Merlot. This may suggest a more expressive fruity profile in this variety, aligning with the higher levels of other acetate esters, such as ethyl isovalerate (57.57 ± 8.33 µg/L in Feteasca neagra), contributing to sweet, fruity, and slightly cheesy notes [93].

N-hexyl acetate, generally associated with green, apple-like aromas [83], had low levels in all samples, the highest value being observed in Pinot noir (1.02 ± 0.02 µg/L), potentially increasing freshness.

An important difference was evidenced in ethyl cinnamate, a compound with cinnamon, sweet spice, and honey aromas [97]. It has significantly high values in Pinot noir (125.12 ± 10.17 µg/L) compared to Merlot (7.88 ± 0.22 µg/L) and Feteasca neagra (36.12 ± 4.82 µg/L). This may highlight varietal specificity or fermentation circumstances promoting aromatic spice development in Pinot noir.

Diethyl succinate, known for its mild fruity and caramel bouquet [97,98], was most abundant in Pinot noir (5031.33 ± 53.21 µg/L), followed by Feteasca neagra and Merlot. This ester usually enhances smoothness and aromatic roundness, aligning with the elevated levels of other esters in Pinot noir.

Methyl-4-hydroxybutanoate, a less common ester with lactone-like, strawberry and banana aromas [82], had elevated value Pinot noir (402.52 ± 46.11 µg/L), possibly enhancing textural perception and aroma complexity.

**Figure 3:**
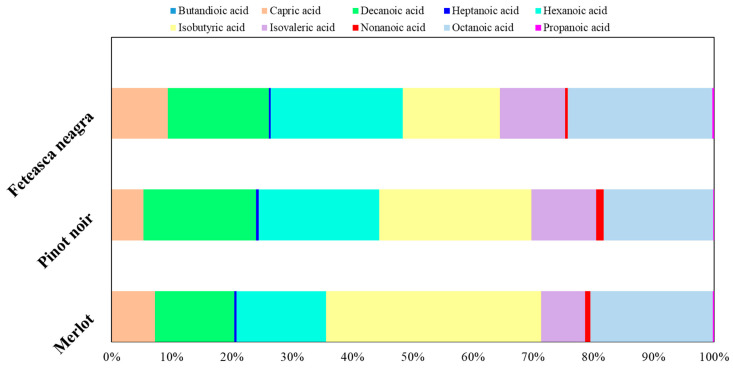
Fatty acids’ compositional profiles (%).

The concentration of various fatty acids in Merlot, Pinot noir, and Feteasca neagra wines exhibited significant variability, reflecting distinct metabolic and fermentation profiles among the cultivars. Total fatty acid concentrations ranged from 5984.75 µg/L in Merlot, followed by Feteasca neagra (5897.93 µg/L) and Pinot noir (5516.32 µg/L) (Figure 3).

Hexanoic acid was predominant across all three wine types, with Feteasca neagra having the highest concentration (1291.87 ± 57.73 µg/L), followed by Pinot noir (1104.11 ± 53.91 µg/L) and Merlot (892.91 ± 55.22 µg/L). This fatty acid contributes to the complexity of wine bouquet, often imparting sweat and fatty sensory notes, which may influence the overall organoleptic profile [88].

Octanoic acid had the highest value in Feteasca neagra (1416.55 ± 65.66 µg/L), slightly exceeding its levels in Merlot (1213.45 ± 66.39 µg/L) and Pinot noir (1003.41 ± 61.78 µg/L). The elevated level in Feteasca neagra may suggest an increase in wine flavor intensity, this compound being linked with acid and oily notes that can modulate wine mouthfeel [83].

Decanoic acid level was the highest in Pinot noir (1033.11 ± 25.13 µg/L) compared to Merlot (786.48 ± 23.01 µg/L). Feteasca neagra revealed average levels (989.88 ± 22.95 µg/L). This fatty acid plays an important role in wine stability and aroma complexity, often contributing to rancid and fatty notes [87].

Isobutyric acid presented an increased concentration in Merlot (2133.43 ± 33.16 µg/L) compared with Pinot noir (1391.22 ± 25.62 µg/L) or Feteasca neagra (951.14 ± 21.74 µg/L). It is often related to unpleasant cheesy or rancid odor at high levels, its abundance in Merlot potentially influencing sensory perception [87].

Isovaleric acid concentrations were elevated in Pinot noir (594.21 ± 20.81 µg/L) and Feteasca neagra (638.02 ± 21.19 µg/L) compared to Merlot (435.92 ± 24.44 µg/L), evidencing potential varietal differences in metabolic mechanisms related to branched-chain fatty acid synthesis [87].

Other acids, such as capric acid and butandioic acid, varied among the red wine samples, with capric acid being the highest in Feteasca neagra (544.31 ± 27.56 µg/L) and lowest in Pinot noir (287.39 ± 24.11 µg/L), contributing to subtle aroma and mouthfeel modifications.

Overall, these differences in fatty acid composition evidenced varietal-specific metabolic pathways during fermentation, contributing to the unique sensory profiles and quality characteristics of each wine. The quantification of fatty acids may provide an insight into the biochemical fingerprint of these cultivars and their potential impact on wine flavor and stability.

**Figure 4:**
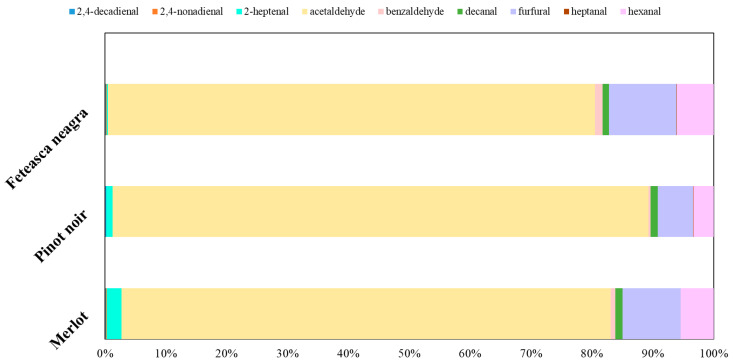
Carbonylic compounds’ compositional profiles (%).

The aldehyde profile of Merlot, Pinot noir, and Feteasca neagra wines evidenced notable variations, indicating distinct biochemical and oxidative processes during winemaking and aging. Pinot noir wine exhibited the most significant concentrations of aldehydes and ketones (8871.79 µg/L), followed by Feteasca neagra (8325.83 µg/L) and Merlot (6878.62 µg/L), with acetaldehyde being the most abundant compound (Figure 4).

Acetaldehyde was the most abundant across all samples, the highest concentration being observed in Pinot noir (7814.23 ± 63.97 µg/L), followed by Feteasca neagra (6662.19 ± 61.99 µg/L) and Merlot (5527.81 ± 62.15 µg/L). Considering the acetaldehyde’s pivotal contribution to wine aroma and its influence on the perception of freshness or oxidation [87], these differences reflect varietal-specific fermentation dynamics and oxidation levels.

Hexanal, a key marker of lipid oxidation and a contributor to green, grassy notes [99], showed the highest concentration in Feteasca neagra (502.23 ± 35.81 µg/L), surpassing levels from Merlot (372.24 ± 34.29 µg/L) and Pinot noir (296.53 ± 25.94 µg/L). This elevated hexanal content in Feteasca neagra may affect its sensory profile by imparting fresh or herbaceous notes.

Furfural concentrations were elevated in Feteasca neagra (922.73 ± 55.55 µg/L) compared to Merlot (657.22 ± 41.05 µg/L) and Pinot noir (509.66 ± 43.11 µg/L). Furfural is known for its sweet, caramel-like fragrance and usually results from Maillard reactions and thermal degradation of sugars, suggesting potential differences in aging or vinification processes [89].

Benzaldehyde, contributing to almond-like aromas [90], was significantly increased in Feteasca neagra (106.03 ± 13.27 µg/L) compared to Merlot (56.73 ± 5.33 µg/L) and Pinot noir (26.35 ± 5.99 µg/L), indicating a distinctive aromatic complexity.

Other aldehydes, such as 2-heptenal, exhibited the highest concentration in Merlot (168.99 ± 5.11 µg/L), exceeding concentration in Pinot noir (89.94 ± 4.88 µg/L) and Feteasca neagra (16.91 ± 7.52 µg/L). It is usually linked with fatty and green odors and may contribute to the varietal character of Merlot [100].

The 2,4-decadienal and 2,4-nonadienal, offering fatty and citrus-like aromas [99,101], displayed variable levels, with 2,4-decadienal being the highest in Pinot noir (18.16 ± 6.11 µg/L) and 2,4-nonadienal the most abundant in Feteasca neagra (9.19 ± 2.33 µg/L). These variations may induce differences in lipid oxidation mechanisms and may influence the nuanced aromatic distinctions among the varieties.

Overall, the carbonylic compounds profiles evidenced the complexity of the oxidative and fermentative pathways, shaping the aroma profiles of these wines, evidencing varietal differences that are important for sensory quality and consumer preference.

**Figure 5:**
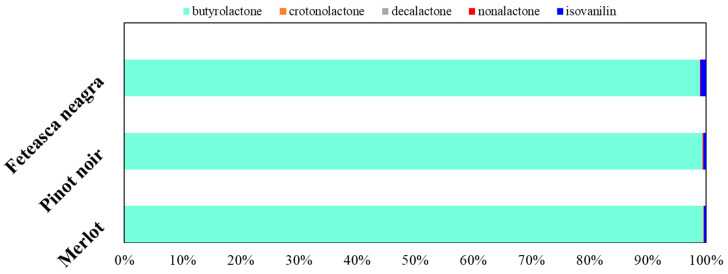
Lactones and other phenolics’ compositional profiles (%).

The concentrations of lactones and related aroma compounds exhibited distinct variations among the three wine varieties. Lactones were identified at significant levels, particularly in Pinot noir wine (9064.99 µg/L), exceeding Merlot (5706.11 µg/L) and Feteasca neagra (6748.45 µg/L) by 32–37% (Figure 5).

Butyrolactone, known for its sweet, plum-like, and creamy aroma [102], was detected in all samples, with Pinot noir having the highest concentration (9001.28 ± 62.99 µg/L), followed by Feteasca neagra (6677.99 ± 45.92 µg/L) and Merlot (5678.66 ± 44.22 µg/L). Its increased level in Pinot noir may be responsible for a more pronounced sweet and round sensory. Crotonolactone, responsible for herbaceous and green notes, was detected only in Merlot (2.35 ± 0.15 µg/L), suggesting a potential varietal specificity or different fermentation dynamics. Decalactone, associated with peach and sweet notes [103], was detected only in Pinot noir (15.54 ± 1.52 µg/L), evidencing a unique lactone profile that may augment the fruity and smooth characteristics. Nonalactone, generating coconut and peach aroma [98], had a descending trend from Merlot (7.28 ± 0.45 µg/L) to Pinot noir (5.06 ± 1.01 µg/L) and Feteasca neagra (2.63 ± 0.99 µg/L), suggesting an increased potential for oak-like aroma contributions in Merlot.

Among other phenolic derivatives, isovanillin, recognized for its sweet, vanilla-like, and slightly smoky notes [104], was detected in all samples, with the highest concentration in Feteasca neagra (67.83 ± 12.54 µg/L), followed by Pinot noir (43.11 ± 11.07 µg/L) and Merlot (17.82 ± 2.21 µg/L). The elevated levels in Feteasca neagra may influence its aromatic depth, contributing to a more complex sensory perception.

Total quantified volatile compounds ranged from 99,018.57 µg/L in Pinot noir to 81,320.35 µg/L in Feteasca neagra, reflecting their distinct aromatic qualities.

Pinot noir exhibited the highest concentration of 2-phenylethanol, contributing to its distinct floral aroma, whereas Feteasca neagra was characterized by elevated levels of 1-hexanol, imparting pronounced herbaceous notes. Ethyl lactate and ethyl acetate predominated in Pinot noir, with higher concentrations conferring fruity and lactic nuances. Volatile fatty acids in Feteasca neagra were distinguished by elevated concentrations of hexanoic acid and octanoic acid, enhancing gustatory complexity. Pinot noir displayed the highest acetaldehyde concentration, associated with fresh, green apple-like characteristics, while benzaldehyde predominates in Feteasca neagra, introducing almond-like undertones.

Principal Component 1 (PC1) accounted for the largest proportion of total variance, primarily reflecting differences in alcohols, esters, and aldehydes/ketones, while Principal Component 2 (PC2) explained a smaller percentage of variance, associated with secondary metabolites and less abundant volatiles. The resulting PCA biplots highlighted both the separation of wine samples according to varietal typicity and the contribution of specific compounds (e.g., 2-phenylethanol, ethyl acetate, butyrolactone) to their differentiation. This multivariate approach not only validated the trends observed in univariate analyses but also provided a holistic overview of the biochemical markers driving varietal distinctions, thereby strengthening the interpretation of the oenological and sensory implications of the studied wines.

**Figure 6:**
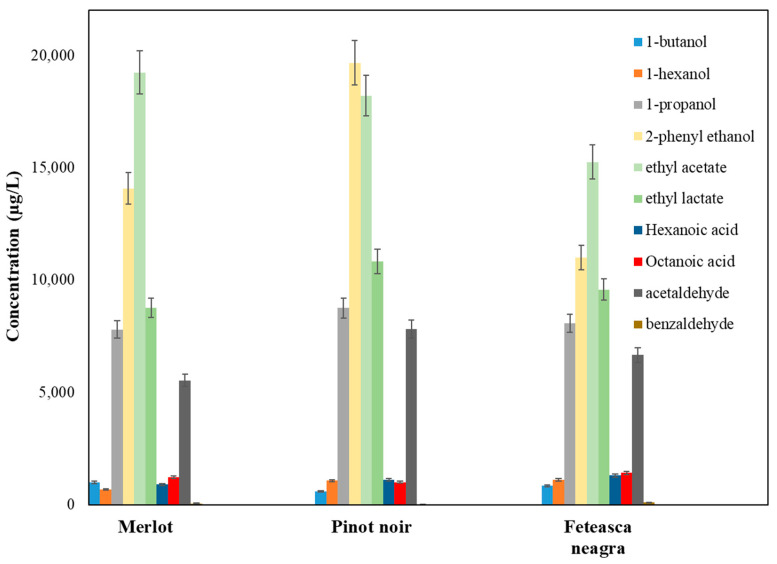
Concentrations of principal volatile compounds across red wine cultivars (Merlot, Pinot noir, Feteasca neagra from Tarnave Vineyard, Romania).

Statistically, Pinot noir exhibited the highest mean concentration of volatile compounds, followed by Merlot and Feteasca neagra, with significant variability (high standard deviations) indicating substantial diversity both between and within compound classes. The concentration ranged from a minimum to a maximum value exceeding 19,000 µg/L (ethyl acetate in Merlot and Pinot noir). ANOVA results for each compound class revealed *p* > 0.05 for all classes, suggesting no statistically significant differences between the wine cultivars (Merlot, Pinot noir, Feteasca neagra) (Figure 6).

Figure 6 presents the comparative concentrations of major volatile compounds in Merlot, Pinot noir, and Feteasca neagra wines. Merlot displayed the highest concentrations of ethyl acetate and higher alcohols, consistent with its fruity and floral aromatic profile. Pinot noir was distinguished by elevated levels of 2-phenylethanol and ethyl lactate, compounds contributing to its characteristic floral and lactic notes. Feteasca Neagrăa showed relatively higher amounts of butyrolactone and isopentyl alcohol, associated with sweet and creamy nuances. These compositional differences underline the role of volatile compounds in defining the varietal typicity of wines from the Tarnave Vineyard.

**Figure 7:**
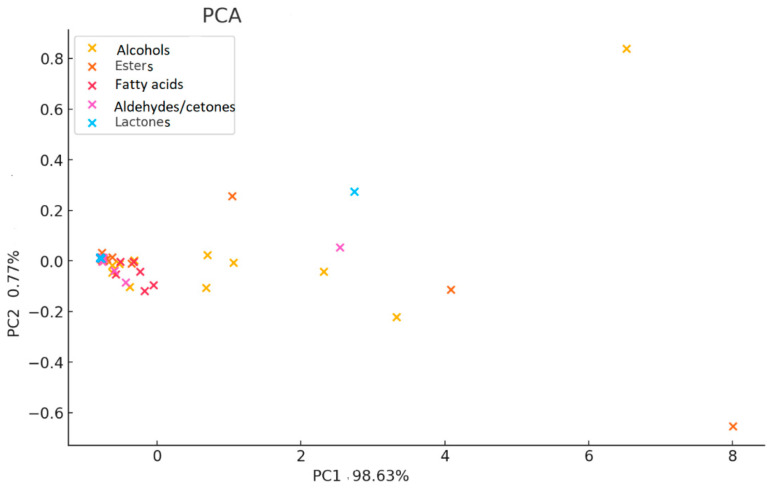
Relationships between volatile compounds and wine Merlot, Pinot noir, Feteasca neagra from Tarnave Vineyard, Romania through principal component analysis (PCA).

Figure 7 clearly indicates that Principal Component 1 (PC1) is the dominant factor in the dataset variance, explaining an exceptional 98.63% of the total variance. This means that the majority of the chemical differences between the wine varieties can be explained by a single dimension.

Principal Component 2 (PC2) accounts for a minor 0.77% of the variance, serving as a secondary, less significant axis of discrimination. Key Drivers of Discrimination The positions of the compounds on the plot reflected their influence on the principal components. The compounds located furthest from the origin, mainly along the PC1 axis, were the most influential in differentiating the samples. The most prominent outliers were several alcohols (orange ‘x’ symbols) located at the far right of the plot (e.g., at PC1 values of 6 and 8). Based on the previously analyzed data, these outliers correspond to compounds like 2-phenyl ethanol, 2-pentanol, and 3-methyl 1-butanol, which were found at significantly higher concentrations in Pinot noir compared to Merlot and Feteasca neagra. Their high positive loadings on PC1 confirmed that they were the primary chemical markers responsible for the separation of the Pinot noir samples. A large cluster of compounds, including most of the fatty acids (red ‘x’) and Lactones (cyan ‘x’), and many esters (light orange ‘x’), were tightly grouped near the origin. This position indicated that these compounds had low loadings on both PC1 and PC2, meaning their concentrations were either relatively similar across all three wine varieties or they did not contribute significantly to the main sources of chemical variation.

The PCA successfully provided a clear visual separation of the three wine varieties, indicating that their volatile compound profiles are sufficiently distinct to serve as chemical markers for varietal differentiation. The analysis showed that PC1 explained 98.63% of the total variance. While this is unusually high, it is a direct result of the large concentration differences of a few key compounds, such as 2-phenyl ethanol and ethyl acetate, among the samples. This finding is not a methodological artifact, but rather a powerful result that indicates the chemical differences between the varietals are overwhelmingly driven by a small number of highly influential volatile compounds, which are the primary determinants of the varietal-specific chemical fingerprints.

This loading plot provided a critical visual confirmation of the initial data analysis, demonstrating that the vast majority of the chemical differences between the three wine varieties can be attributed to a small number of specific compounds, predominantly certain alcohols. The plot effectively visualizes that while all compounds are part of the wine matrix, only a select few are powerful discriminators, with their high loadings on PC1 explaining the distinct clustering observed in the score plot.

Statistically significant correlations between total polyphenols, anthocyanins, and antioxidant capacity further validated the bio-functional potential of these red wines. While this analysis establishes the foundational relationships, a more detailed multivariate analysis would be a valuable direction for future research to elucidate the complex network of relationships among these bioactive compounds.

**Figure 8:**
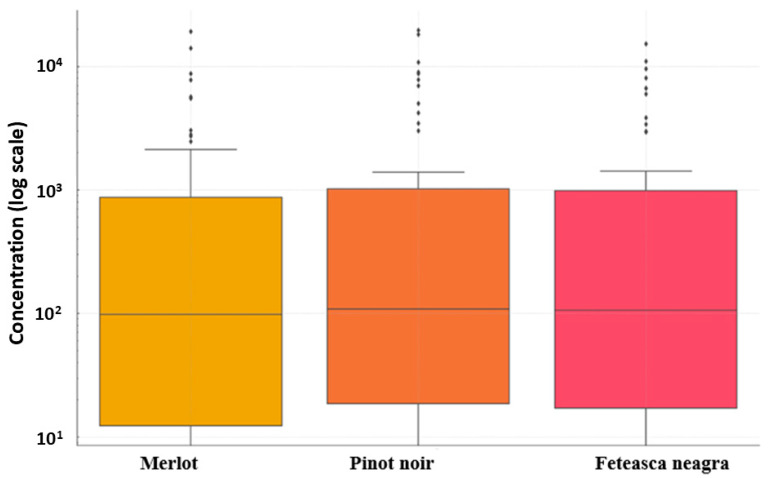
Distribution of volatile compound concentrations for the three wine cultivars (Merlot, Pinot noir, Feteasca neagra from Tarnave Vineyard, Romania).

Figure 8 illustrates the distribution of volatile compound concentrations across the wine varietals (Merlot, Pinot noir, and Feteasca neagra). Logarithmic axes in the box plot enabled visualization of compound concentrations, revealing that Pinot noir exhibited greater variability for most compounds, indicator of higher chemical complexity. Feteasca neagra displayed elevated concentrations of higher alcohols, while Merlot showed a more uniform distribution and intermediate concentrations for most compounds.

The highest absolute mean differences were observed for 2-phenylethanol (mean difference: 5781.17 µg/L), a significant contributor to distinct floral aromas; ethyl acetate (mean difference: 2653.25 µg/L), influencing fruity and volatile aromatic profiles; butyrolactone and diethyl succinate (mean differences: 2215.08 µg/L and 1530.45 µg/L, respectively), associated with sweet, creamy notes and aromatic complexity; acetaldehyde (mean difference: 1524.28 µg/L), critical for light oxidative character; ethyl lactate (mean difference: 1378.63 µg/L), modulating sweet and lactic nuances; 2-pentanol (mean difference: 885.55 µg/L), contributing to alcoholic and floral traits; isobutyric acid (mean difference: 788.19 µg/L), affecting gustatory balance; 3-methyl-1-butanol (mean difference: 779.67 µg/L), enhancing aromatic intensity; 2,3-butanediol (mean difference: 663.55 µg/L), influencing texture and complexity.

### 2.4. Sensory Implications and Varietal Typicity

The quantitative and qualitative distinctions between Merlot, Pinot noir and Feteasca neagra from Tarnave Vineyard highlighted the biochemical diversity among varietals, shaping their sensory identity and perceived complexity.

In terms of volatiles, the three wine varietals exhibited distinct chemical fingerprints. Pinot noir was characterized by the highest concentrations of total volatile compounds, with particular richness in higher alcohols, esters, aldehydes, ketones, and lactones. This quantitative profile suggests a complex aromatic and organoleptic bouquet with a pronounced fruity, floral, and sweet character. Feteasca neagra, while rich in higher alcohols and volatile fatty acids, presented lower levels of esters and aldehydes, which may contribute to a more restrained aromatic profile. Nevertheless, its volatile matrix resulted in a nuanced sensory expression marked by vegetal, creamy, and vanilla-like notes. Merlot demonstrated a balanced volatile profile, characterized by increased concentrations of volatile fatty acids, which enhance mouthfeel and roundness, complemented by moderate ester levels and a perceptible freshness. It must be stated that the sensory impact of these compounds is not solely determined by their concentration, but also by their individual olfactory thresholds. A more detailed analysis of these compounds relative to their sensory detection limits would be a valuable subject for future sensory studies to more precisely correlate the chemical data with the perceived specific aromas.

Pinot noir was differentiated by its multi-aromatic and organoleptic complexity, driven by varietal-specific biochemical mechanisms. The wine exhibited the highest concentrations of 2-phenylethanol, a compound essential for its intense floral (rose-like) aroma [105,106], as well as ethyl lactate and ethyl acetate, conferring sweet, fruity, and lactic notes [20]. The dominance of butyrolactone evidenced its role in coconut flavor and sweetness [27], further enhanced by high level of 2,3-butanediol, contributing to bitter taste [107] and serving as a sweet aroma marker [18].

Pinot noir oxidative character was attributed to the acetaldehyde concentration, while its higher total esters accumulation amplified aromatic intensity [22], distinguishing itself from the other varieties. The wine anthocyanin-rich profile offers chromatic stability and resistance to oxidative browning, aligning with its reputation for age-worthiness [108]. Aromatic complexity can also be enriched by ethyl cinnamate, which imparts cinnamon and dried fruit nuances [16,109]. Despite high intra-class variability in volatile compounds, the biochemical richness of Pinot noir (99,018.57 µg/L total volatile compounds) may reflect its metabolic adaptability under climatic conditions, yielding a sensory identity marked by a flavor balance.

Feteasca neagra was characterized by a vegetal aromatic profile and organoleptic complexity, shaped by its unique metabolic pathway. Isovanillin gave vanilla and spicy notes, while elevated 1-hexanol resulted in amplified herbaceous notes [110], while benzaldehyde and hexanal provided almond-like undertones [111] and fresh grass aromas, respectively [25]. The ethyl hexanoate and ethyl butanoate levels contributed to tropical fruit and apple-like notes, differentiating its aromatic profile [112]. The wine’s average volatile compound concentrations (81,320.35 µg/L total volatile compounds) reflected a balanced biochemical pathway, prioritizing aromatic intensity over sheer abundance, with terroir-driven geo-specificity.

Merlot demonstrated a balanced sensory profile, marked by tartness and organoleptic harmony. The high level of isobutyric acid imprinted sweet-sour notes, while 1-butanol yielded to dried fruit nuances, creating a contrast between acidity and sweetness [22]. The uniform distribution of volatile fatty acids (5984.75 µg/L total) and average polyphenol concentrations reflected a metabolic prioritization of balance over extremes. The ethyl acetate levels (19,234.01 µg/L) enhanced fruity volatility, though its lower lactone concentrations resulted in a less pronounced vanilla character compared to Pinot noir [113]. The varietal moderate antioxidant activity (IC_50_ = 228.12 µg/mL) and stable phenolic matrix aligned with its subtler oxidative aging potential, pleasing to consumers which prefer freshness over prolonged complexity. Merlot agroclimatic adaptability under rising temperatures was evident in its consistent biochemical output, with thermal accumulation and solar radiation trends optimizing sugar-to-acid balance and phenolic maturity, reinforcing its market-ready typicity.

Table 3 provides a summary of key aroma-active compounds found in the three varieties of red wines (Feteasca neagra, Merlot, and Pinot noir), classified by chemical group, highlighting their sensory descriptors and referencing studies published in peer-reviewed journals. The volatile markers identified within this study may be corroborated with subsequent analysis, such as isotopic fingerprinting and multi-element evaluation, to construct a more accurate representation of the geographical origin of the wines under investigation.

The opposite relationship between IC_50_ values and antioxidant efficacy confirmed methodological coherence across DPPH, FRAP, and TEAC assays, while the statistically significant positive correlation (*p* < 0.05) between total polyphenol content and antioxidant capacity underscored the redox-active role of phenolic hydroxyl groups.

ANOVA results for principal volatile compound classes across Merlot, Pinot noir, and Feteasca neagra wines yielded non-significant differences (*p* > 0.05), most probably attributed to high intra-class variability within compound groups, which obscured inter-cultivar distinctions. This variability, compounded by dataset limitations (e.g., sample size, unaccounted confounding variables such as microclimatic fluctuations or vinification practices), suggests that cultivar-specific differences in volatile compound classes are subtle or context-dependent. Principal component analysis (PCA) further elucidates these relationships, with Principal Component 1 (PC1) explaining 98.63% of total variance, primarily driven by compound abundance rather than class-specific clustering. In contrast, PC2 accounts for only 0.77% variance, underscoring the shared biochemical pathways influencing aroma and flavor across varietals.

Outliers identified in Figure 8, such as acetaldehyde in Pinot noir (7814.23 µg/L) and benzaldehyde in Feteasca neagra (106.03 µg/L), may define specific wines, though their contributions remain secondary to abundance-driven differentiation.

Box plot analysis (Figure 8) of volatile compound distributions, employing logarithmic axes, revealed that Pinot noir had increased biochemical variability, aligning with its multifaceted aromatic profile. Mean differences in key compounds, such as 2-phenylethanol, ethyl acetate, and butyrolactone, emerge as biomarkers for varietal distinction, though their sensory impacts are modulated by overlapping metabolic pathways. These findings, consistent with global studies [2,118], emphasize the robustness of observed trends despite methodological or regional disparities in absolute concentrations.

Overall, the volatile compound profiles of the three varietals highlighted different aromatic fingerprints, determined by both intrinsic varietal features and extrinsic factors (fermentation and maturation protocols). The rich esters and lactones content of Pinot noir evidenced its fruit-forward sensory persuasiveness, while the chemical robustness of Feteasca neagra exhibited its potential to yield more intense and structured wines. Merlot, having a harmonious composition, may be a versatile varietal suitable for broader consumer choices. It must be highlighted that these aroma characteristics are postulated using the chemical data. A formal sensory evaluation, such as one involving a trained panel or a consumer test, would be a valuable direction for future research to directly validate these findings and provide a comprehensive understanding of the perceived sensory profiles.

## 3. Materials and Methods

### 3.1. Reference Zone

The Tarnave Vineyard, one of Romania’s most renowned wine-growing regions, is celebrated for its premium white wines. Situated within the hydrographic basin of the Tarnava Mare and Tarnava Mica rivers, it spans latitudes 45°57′ to 46°32′ N and longitudes 23°52′ to 24°48′ E. The hilly terrain positions vineyards predominantly on sun-exposed slopes (south, southeast, southwest) or in inter-hill valleys, which provide natural protection against wind and frost. Vineyards are located at altitudes exceeding 250 m, with slopes of 5–20%, and thrive on a diverse pedological mosaic, including brown soils, sandstone, and clay-sandstone substrates. The region is further characterized by brown-eumezobasic soils and clay-illuvial browns, which are known for their heat and moisture retention properties, crucial for viticulture in this area.

Hydrologically, the region is fed by deep aquifers and hillside springs. The climate, critical for quality yields, features moderate summers, wet and harsher winters, and long, clear autumns, with an annual mean temperature of 9.5–9.8 °C. Current temperatures are 1–1.5 °C higher than the 1950–2023 average. During grape ripening, daytime temperatures now average 22 °C, while nighttime temperatures average 12 °C (vs. 9.09% lower historically) according to the Archive of the National Meteorological Administration of Romania [119]. Persistent fog in late summer and early autumn slows ripening, with relative humidity averaging 70%.

### 3.2. Evaluation of Climatic Parameters for Vineyard Management and Regional Climate Zoning

Oenoclimatic parameters included:

- Global radiation (MJ/m^2^/day)*,* measured via pyranometer during the growing season (1 April–30 September).
- Effective temperature sum (ƩT_e_, °C), calculated as the cumulative daily temperature increments exceeding the 10 °C biological threshold for vine development during the growing season:

(1) $$\sum T_{e}=\left(T_{m}-10\right)\times N_{c}$$

where T_m_ = daily mean temperature (°C), N_c_ = number of days within the 1 April–30 September period, and 10 = biological threshold temperature (°C) below which *Vitis vinifera* L. ceases physiological activity.

Real heliothermal index (RHI) was calculated as:

(2) $$\mathrm{RHI}=\sum \frac{T_{m}-10}{1+0.1\times \left(T_{M}-T_{m}\right)}\;$$

where T_m_ = daily minimum temperature (°C) and T_M_ = daily maximum temperature (°C), reflects heliothermal resource quality during the growing season [120].

Bioclimatic index (I_bcv_) assesses regional climatic suitability for viticulture [121] and was calculated as:

(3) $$I_{\mathrm{bcv}}=\sum \frac{T_{m}-10}{1+0.1\times T_{M}}\;$$

The hydro-thermal coefficient of Selyaninov (HTC) was employed to quantify the precipitation-to-temperature ratio, functioning as a critical agroclimatic indicator of water availability in viticulture. HTC integrates hydrological and thermal variables to assess climatic suitability for grapevine cultivation, offering a robust metric for evaluating irrigation demands and drought stress under shifting climatic conditions. This index was calculated as:

(4) $$\mathrm{HTC}=\frac{P}{0.1\times \sum T_{m}}$$

where P = total precipitation (mm) and ΣT_m_ = sum of monthly mean temperatures (°C) during the growing season [3].

A scaling coefficient of 0.1 was applied to standardize the derived parameters, ensuring their transformation into readily interpretable values within the analytical framework.

The De Martonne aridity index (DMI) was applied to quantify the precipitation-to-temperature ratio [54], and it was calculated as:

DMI = PT + 10I = T + 10P

where P is annual precipitation in mm and T is mean annual temperature in °C.

This index serves as a critical agroclimatic metric for classifying the climatic suitability of viticultural regions, enabling the assessment of water availability and aridity thresholds essential for sustainable grapevine cultivation.

Oenoclimatic aptitude index (IAOe) is a composite agroclimatic metric designed to evaluate the viticultural potential of a region by integrating key climatic variables, specifically temperature, precipitation, and solar radiation. The index was computed as:

(5) $$\mathrm{IAOe}=\frac{\mathrm{HI}}{2}+\;\frac{Q}{20}+10-\frac{P}{200}$$

where HI = Huglin Heliothermic index (°C), which quantifies heat accumulation during the growing season; Q = cumulative global radiation over the vegetative period (MJ/m^2^); and P = total precipitation (mm) during the vegetative period [122].

This index synthesizes thermal energy, photosynthetic potential, and hydrological constraints to classify regions based on their suitability for *Vitis vinifera* L. cultivation. Higher IAOe values correlate with enhanced climatic favourability for viticulture, balancing heat-driven maturation, solar resource availability, and water stress mitigation.

The Winkler Index, along with the Real Heliothermal Index (RHI), were selected as they are globally recognized metrics that quantify the complex interaction of temperature and solar radiation on grapevine physiology and fruit maturation. Complementary to these, the hydro-thermal coefficient of Selyaninov (HTC) and the De Martonne aridity index (DMI) were utilized to assess water balance, a crucial factor for grape quality in a region experiencing climatic shifts. This multi-metric approach provides a more comprehensive understanding of the vineyard-specific terroir.

### 3.3. Reagents and Equipment

All reagents, including Folin–Ciocalteu reagent, anhydrous sodium carbonate, gallic acid, DPPH (1,1-diphenyl-2-picrylhydrazyl), Trolox (6-hydroxy-2,5,7,8-tetramethylchroman-2-carboxylic acid), 2,4,6-tripyridyl-s-triazine (TPTZ), ferric chloride, hydrochloric acid, methanol, disodium phosphate, citric acid, potassium chloride, sodium acetate, potassium persulfate, ABTS (2,2′-azinobis(3-ethylbenzothiazoline-6-sulfonic acid)), sodium chloride, dichloromethane, anhydrous sodium sulfate, and ethanol, were of analytical grade and purchased from Sigma-Aldrich GmbH (Steinheim, Germany).

Analytical instrumentation comprised a UV-1900 SHIMADZU spectrophotometer (Shimadzu Corporation, Kyoto, Japan) for absorbance measurements and a Nexis GC-2030 GCMS-TQ8050NX gas chromatograph–mass spectrometer (Shimadzu, Kyoto, Japan) equipped with a triple quadrupole mass detector for chromatographic separations and compound identification.

### 3.4. Determination of Total Polyphenols (Folin-Ciocâlteu Method)

Total polyphenols were quantified spectrophotometrically using the Folin–Ciocalteu method [2]. Samples of red wine were diluted 1:20 with distilled water. A 0.25 mL aliquot was then mixed with 1.25 Ml of Folin–Ciocalteu reagent. After 5 min, 7.5% sodium carbonate solution (1 mL) was added. The mixture was incubated in the dark at room temperature for 60 min, after which absorbance was measured at 765 nm using a spectrophotometer. Results were reported as milligrams of gallic acid equivalents per liter (mg GAE/L), the calibration curve being designed using gallic acid standards.

### 3.5. Total Anthocyanins Assessment (pH Differential Method)

Total anthocyanins were estimated through a pH differential spectrophotometric method [2], in which the absorbance was measured at two distinct pH values (1.0 and 4.5) and two wavelengths (515 nm and 700 nm). Samples were normalized using two buffering solutions (0.025 M KCl for pH 1.0 and 0.4 M sodium acetate trihydrate for 4.5), then incubated at ambient temperature for half an hour. Absorbance (A) was calculated as:

A = (Abs_515_ − Abs_700_)_pH1.0_ − (Abs_515_ − Abs_700_)_pH4.5_ (6)

where Abs_515_ and Abs_700_ represent absorbance values at the respective wavelengths for each pH condition.

Total anthocyanin concentration was obtained as follows:

Anthocyanin (mg/L) = A × MW × DF/ε × l,(7)

where A = absorbance, MW (molecular weight) = 493.5 g/mol for malvidin-3-glucoside, 449.2 g/mol for cyanidin-3-glucoside, DF = dilution factor, *l* = path length of the cuvette (1 cm), and *ε* = molar extinction coefficient (26,900 L·mol^−1^·cm^−1^).

### 3.6. Antioxidant Activity Assessment (DPPH, TEAC, FRAP)

#### 3.6.1. DPPH Radical Scavenging Assay

The antioxidant capacity was assessed using a slightly modified DPPH radical scavenging method [2]. A methanolic DPPH solution (80 μM) was prepared and stored at 4 °C. 250 μL wine sample was mixed with 1750 μL of DPPH solution and incubated in the dark for half an hour. The blank was obtained by replacing the sample with methanol. Absorbance was measured spectrophotometrically at 517 nm. The percentage of DPPH radical scavenging activity was calculated as:

(8) $$\mathrm{DPPH}\;(\%)\;=\;\frac{\mathrm{Ab}-\mathrm{Aa}}{\mathrm{Ab}}\times 100$$

where *Ab* and *Aa* represent the absorbance of the blank and the absorbance of the sample, respectively. Results were standardized against a Trolox calibration curve and expressed as millimolar Trolox equivalents per liter (mmol TE/L).

The IC_50_ value was defined as the concentration of wine extract (µg/mL dry extract equivalent) required to inhibit 50% of DPPH radical activity. Although 250 µL of wine sample was added to the reaction mixture, the concentrations were recalculated and expressed as µg/mL dry extract equivalent in order to normalize results and allow comparison with literature data. IC_50_ values were determined from the linear regression of inhibition percentage against concentration.

#### 3.6.2. Trolox Equivalent Antioxidant Capacity (TEAC)

The TEAC assay is usually used to evaluate the antioxidant activity through the synthetic radical designated as ABTS+ (cation radical of 2,2′-azino-bis), generated by oxidizing ABTS with potassium persulfate [2]. The reaction mixture was incubated at 22 °C for 12 h in the dark. The ABTS+ was diluted to an absorbance of 0.70 at 734 nm and mixed with 20 μL of Trolox standards (0–25 μM). After a 6-min reaction at room temperature, absorbance was measured at 734 nm. Antioxidant capacity was expressed in micromolar or millimolar Trolox equivalents per liter (μmol TE/L or mmol TE/L).

#### 3.6.3. Ferric Reducing Antioxidant Power (FRAP)

The FRAP assay evaluates the total antioxidant capacity via the reduction of ferric ions (Fe^3+^) to ferrous state (Fe^2+^), in the presence of 2,4,6-tri(2-pyridyl)-s-triazine (TPTZ). 50 mL acetate buffer (pH 3.6), 5 mL FeCl_3_ (20 mM), and 5 mL TPTZ (10 mM, pre-acidified with 150 μL HCl) were mixed to obtain FRAPT reagent. 5 mL FRAP reagent reacted with 1 mL sample and 20 mL distilled water, followed by incubation in the dark at 20 °C for 1 h. Absorbance was measured at 595 nm. The results were expressed as micromolar or millimolar Trolox equivalents per liter (μmol TE/L or mmol TE/L).

### 3.7. GC–MS Determination of Aroma Compounds

The GC–MS determination was adapted from a previous developed procedure [123]. Prior to analysis, the wines were stored at 4 °C ± 0.5 °C. To isolate VOCs, a liquid–liquid microextraction procedure was used: 8 mL sample were mixed with 2.5 g sodium chloride (NaCl) and 4 mL dichloromethane (DCM), mixing at 2500 rpm for 3 min and subsequent immersing in an ice bath for 5 min. The mixture was then centrifuged at 3000 rpm for 3 min. A 2 mL aliquot of the organic supernatant was combined with 0.5 g anhydrous sodium sulfate (Na_2_SO_4_) to remove residual water, centrifuged again under identical conditions, and filtered through a 0.45 μm polytetrafluoroethylene (PTFE) membrane. The extract was transferred to gas chromatography vials. The extraction procedure was repeated five times to ensure analytical precision.

Volatile compounds were analyzed using a triple quadrupole gas chromatography–mass spectrometry system equipped with an Rtx-5MS capillary column (30 m × 0.25 mm × 0.25 μm film thickness). The injector temperature was maintained at 220 °C, and 1 μL sample was injected in splitless mode. The carrier gas was helium at a flow rate of 1.4 mL/min. The oven temperature program was initiated at 40 °C, where it was held for 7 min, ramped to 95 °C at 5 °C/min (held for 4 min), then increased to 120 °C at 5 °C/min (held for 7 min), followed by a rapid temperature rise to 280 °C at 55 °C/min (held for 12 min). Volatile compounds were identified by matching their mass spectra against the NIST MS Search 2.4 spectral library (National Institute of Standards and Technology, Gaithersburg, MD, USA).

To determine the quantitative profile of volatile aroma compounds, 20 reagent standards (Sigma-Aldrich GmbH, Steinheim, Germany) were used, covering alcohols, esters, aldehydes, carboxylic acids, and terpenoid compounds. Two stock standard solutions (1000 and 10 ppm) were prepared, from which calibration standards were generated: 5, 20, 50, 100, 200, 500 ppb (µg/L) and 2, 10, 25, 50 ppm (mg/L). Each calibration level was analyzssed in triplicate injections. Within this adapted sample preparation protocol, recovery rates for each analyte were assessed by processing a set of eight identical simulated wine samples (composition: distilled water, 12.5% ethanol, 2 ppm of each standard). The specific elution order of analytes corresponded to their retention indices. Considering the two-fold concentration step during sample preparation (8 mL wine → 4 mL DCM), chromatogram-derived data were subsequently corrected for concentration factors and further adjusted for extraction efficiency from the wine matrix. Recovery of volatile compounds was determined using eight model wine samples (distilled water, 12.5% ethanol, 2 ppm of each standard) processed via the same liquid–liquid microextraction procedure. Known amounts of standards were added to the wine samples, and recovery rates were used to correct the GC–MS data for extraction efficiency and the two-fold concentration factor (8 mL wine → 4 mL DCM).

### 3.8. Statistical Analysis

Statistical analysis was performed to evaluate both univariate and multivariate relationships among the investigated parameters. Differences in polyphenolic content, anthocyanin levels, antioxidant capacity, and volatile compound concentrations across wine varieties were assessed using one-way analysis of variance (ANOVA), followed by post-hoc tests where appropriate, in order to identify statistically significant differences (*p* < 0.05). Regression analyses were conducted to examine linear relationships between antioxidant assays (DPPH, TEAC, FRAP) and polyphenolic/anthocyanin content, thereby elucidating the extent to which phenolic composition contributes to antioxidant potential. To explore multivariate dependencies, principal component analysis (PCA) was applied using XLSTAT Addinsoft 2014.5.03 (Addinsoft Inc., New York, NY, USA). PCA enabled the reduction of high-dimensional data into orthogonal components that summarize the majority of variance while preserving the structure of inter-variable correlations. Volatile compounds were projected onto principal component axes to identify clustering patterns among the three red wine cultivars (Feteasca neagra, Merlot, and Pinot noir).

## 4. Conclusions

The observed upward trends in temperature, solar radiation, and aridity may suggest potential positive effects on grape ripening, including for red varieties. Nevertheless, these climatic shifts highlight the demand for adaptive protocols in vineyard water management to facilitate optimal grape quality under evolving environmental conditions.

The red wines from the studied region exhibited increased levels of polyphenolic compounds, characteristic to their respective cultivars and comparable to those found in wines from established viticultural areas. Among the three varieties, Merlot had the lowest concentrations of antioxidant compounds, which may align with consumer preferences for lighter and more delicate wines. Pinot noir exhibited an average profile, balancing moderate levels of bioactive compounds with sensory finesse, potentially attractive to consumers seeking both health benefits and refined taste. On the contrary, Feteasca neagra evidenced itself as the most chemically and functionally potent variety, with the highest levels of polyphenols, anthocyanins, and antioxidant activity, ensuring the position of candidate for premium wines targeted at health-conscious markets.

Statistically significant correlations between total polyphenols, anthocyanins, and antioxidant capacity further validated the bio-functional potential for these red wines.

In terms of volatile composition, Pinot noir exhibited the highest concentrations of volatile compounds, including higher alcohols, esters, aldehydes, ketones, and lactones. This may indicate a complex aromatic and organoleptic profile with a pronounced fruity, floral, and sweet bouquet. Feteasca neagra, rich in higher alcohols and volatile fatty acids, presented lower levels of esters and aldehydes, leading to a more restrained aromatic profile. However, its volatile matrix resulted in a nuanced expression marked by vegetal, creamy, and vanilla-like notes. Merlot demonstrated a balanced volatile profile, characterized by increased concentrations of volatile fatty acids, enhancing mouthfeel and roundness, balanced by moderate ester levels and a perceptible freshness.

Overall, the volatile compound profiles of the three varietals highlighted different aromatic fingerprints, determined by both intrinsic varietal features and extrinsic factors (fermentation and maturation protocols). The rich esters and lactones content of Pinot noir evidenced its fruit-forward sensory persuasiveness, while the chemical robustness of Feteasca neagra exhibited its potential to yield more intense and structured wines. Merlot, having a harmonious composition, may be a versatile varietal suitable for broader consumer choices.

These findings evidenced the essential role of volatile and phenolic compounds in determining wine typicity and sensory identity. They also deliver a scientific foundation for varietal differentiation, quality assurance, and targeted marketing approaches in the context of changing climatic and consumer preferences. Future investigations will also focus on a detailed analysis of the vineyard’s soil composition to provide a more comprehensive understanding of the terroir effect on the final volatile profile of the wines.

## Figures and Tables

**Table 1:** Climatic characterization of the wine region across five distinct periods.

Indicators	Mean Value of Indicators				
	1950–1963	1964–1978	1979–1993	1994–2008	2009–2023
Global radiation (MJ/m^2^/day)	20.56	21.01	21.09	22.19	22.24
Effective temperature sum	1533.32	1547.88	1539.76	1602.33	1612.47
Real heliothermal index	1.79	1.83	1.88	1.89	2.01
bioclimatic index	7.42	7.48	8.02	8.93	9.12
Hydro-thermal coefficient of Selyaninov	1.27	1.78	1.77	1.43	1.32
De Martonne aridity index	17.43	15.38	17.17	18.47	19.96
Oenoclimatic aptitude index	29.22	28.36	28.91	30.03	32.67

**Table 2:** Variation in total polyphenols, total anthocyanins, and antioxidant activity in red wine samples of Merlot, Pinot noir, and Feteasca neagra.

Parameters	Wine		
	Merlot	Pinot Noir	Feteasca Neagra
Total polyphenols (mg GAE/L)	2328.34 ± 1.18	2539.19 ± 1.07	2814.22 ± 1.21
Total anthocyanins (mg M3GE/L)	423.32 ± 0.73	477.77 ± 0.84	526.33 ± 0.72
DPPH (%)	68	75	84
IC_50_ (µg/mL)	228.12	196.45	115.32
Antioxidant activity (mmol TE/L)	7.47	9.32	12.92
TEAC (mmol TE/L)	10.18 ± 0.51	12.02 ± 0.29	13.62 ± 1.16
FRAP (mmol TE/L)	10.97 ± 0.83	12.39 ± 0.47	13.45 ± 1.22

Data were expressed as the mean of triplicate measurements ± standard deviation; mg GAE/L = milligrams of gallic acid equivalent per liter; mg M3GE/L = milligrams of malvidin-3-glucoside equivalent per liter; DPPH = 1,1′-Diphenyl-2-picrylhydrazyl radical; IC_50_ (µg/mL) = antioxidant efficiency expressed as the half-maximal inhibitory concentration (µg/mL); mmol TE/L = millimoles of Trolox equivalent per liter; TEAC (mmol TE/L) = Trolox equivalent antioxidant capacity, expressed as millimoles of Trolox equivalent per liter (mmol TE/L); FRAP (mmol TE/L) = Ferric reducing antioxidant power, expressed as millimoles of Trolox equivalent per liter (mmol TE/L).

**Table 3:** Classification of aroma-active compounds in red wine according to chemical class and sensory descriptor.

Class	Compound	Aroma Descriptor	Odor Series *	Reference
Higher alcohols	1-Butanol	Intoxicating aroma, alcoholic odor	1	[83]
	1-Heptanol	Bouquet plant odor, grape odor	6	[83]
	1-Hexanol	Leafy and fruity notes	4	[83]
	1-Propanol	Bouquet, ripe fruity notes	6	[83]
	2,3-Butanediol	–	-	-
	2-Nonanol	Strong fruity notes, rose odor	2	[83]
	2-Pentanol	–	-	-
	2-Phenylethanol	Rose aroma	2	[84]
	3-Methyl-1-butanol	Cheese odor	5	[83]
	3-(Methylthio)-1-propanol	Meaty or onion-like aromas	8	[82]
	Benzyl alcohol	Candy, fruity	3	[85]
	β-Linalool	Floral and citrus-like aromas	2	[91]
	Citronellol	Floral	2	[87]
	(E)-3-Hexen-1-ol	Freshly cut grass	8	[88]
	Furfuryl alcohol	Burned	9	[89]
	Isoamyl alcohol	Fruit aroma	6	[90]
	Linalool	Muscat	2	[91]
	1-Pentanol (n-Amyl alcohol)	Floral	2	[2]
	n-Propanol	Ripe fruit	6	[93]
	α-Terpineol	Floral, candy	2	[85]
	(E)-Geraniol (Trans-Geraniol)	Muscat	2	[91]
	(Z)-3-Hexen-1-ol	Fresh, fruity	6	[95]
Esters	2-Phenylethyl acetate	Floral	2	[96]
	Diethyl succinate	Caramel, fruity	3	[97,98]
	Ethyl acetate	Fruity odor, ester odor	6	[83]
	Ethyl butanoate	Fruity, strawberry	6	[95]
	Ethyl butyrate	Fruity	6	[87]
	Ethyl cinnamate	Honey, cinnamon, floral	3	[97]
	Ethyl decanoate	Caramel, fruity	3	[87]
	Ethyl hexanoate	Green apple aroma, fruity odor	4	[83]
	Ethyl isovalerate	Fruity, apple	6	[93]
	Ethyl lactate	Buttery or subtle fruity aroma	6	[93]
	Ethyl nonanoate	Fruity, floral	6	[85]
	Ethyl octanoate	Fruity odor, caramel	6	[83]
	Isoamyl acetate	Banana and pear-like notes	6	[93,97]
	Methyl 4-hydroxybutanoate	Strawberries, bananas	6	[82]
	n-Hexyl acetate	Pleasant fruity, green apple odor	6	[83]
Volatile fatty acids	Butanedioic acid	–		[18]
	Capric acid	Rancid fat	5	[114]
	Decanoic acid	Rancid fat	5	[87]
	Heptanoic acid	Fatty, dry	5	[115]
	Hexanoic acid	Copra oil, sweat	5	[83]
	Isobutyric acid	Rancid, butter, cheese	5	[87]
	Isovaleric acid	Acidic, rancid	5	[87]
	Nonanoic acid	Fruity, fatty, joyful	6	[116]
	Octanoic acid	Light fruity acid odor	6	[83]
	Propanoic acid	Cheese	5	[117]
Aldehydes and ketones	2,4-Decadienal	Fatty and citrus-like aromas	4	[101]
	2,4-Nonadienal	Fatty and citrus-like aromas	4	[99]
	2-Heptenal	Fatty and green odor	-	[100]
	Acetaldehyde	Pungent, ripe apple	6	[87]
	Benzaldehyde	Almonds	3	[85]
	Decanal	Grassy, orange skin-like	8	[115]
	Furfural	Burned	9	[89]
	Heptanal	–	-	-
	Hexanal	Herbaceous	4	[99]
Lactones	γ-Butyrolactone	Plum aroma	6	[102]
	Crotonolactone	–	-	-
	δ-Decalactone	Fruity, sweet, floral	2	[103]
	γ-Nonalactone	Coconut, peach	6	[93]
	Isovanillin	Sweet, vanilla, and slightly smoky	2	[104]

* Odor series: 1 solvent, 2 floral, 3 sweet, 4 green, 5 buttery, 6 fruits, 7 balsamic, 8 grass, 9 burned, 10 woody [87].

## Data Availability

The original contributions presented in this study are included in the article. Further inquiries can be directed to the corresponding authors.

## Abbreviations

The following abbreviations are used in this manuscript:

DPPH	2,2-diphenyl-1-picrylhydrazyl
TEAC	Trolox Equivalent Antioxidant Capacity
FRAP	Ferric Reducing Antioxidant Potential
GC–MS	Gas Chromatography/Mass Spectrometry
PCA	Principal Component Analysis
PC	Principal Component
VOCs	Volatile Organic Compounds
RHI	Real Heliothermal Index
Ibcv	Bioclimatic Index
HTC	Hydro-Thermal Coefficient of Selyaninov
DMI	The De Martonne Aridity Index
IAOe	Oenoclimatic Aptitude Index
TPTZ	2,4,6-tripyridyl-s-triazine
ABTS	2,2′-azinobis(3-ethylbenzothiazoline-6-sulfonic acid
GAE	Gallic Acid Equivalents
A	Absorbance
MW	Molecular Weight
DF	Dilution Factor
TE	Trolox Equivalents
l	Path Length of the Cuvette
ε	Molar Extinction Coefficient
PTFE	Polytetrafluoroethylene
DCM	Dichloromethane
